# Comparing options for women seeking permanent contraception in high-resource countries: a protocol for a systematic review

**DOI:** 10.1186/s13643-019-0987-7

**Published:** 2019-03-26

**Authors:** Rebecca Gormley, Brian Vickers, Wendy V. Norman

**Affiliations:** 1 Faculty of Health Sciences, Simon Fraser University, Burnaby, Canada; 2 Contraception and Abortion Research Team-Groupe - de recherche sur l’avortement et la contraception (CART-GRAC), BC Women’s Hospital, Vancouver, Canada; 3 Faculty of Medicine, University of British Columbia, Vancouver, Canada; 4 Department of Family Practice, University of British Columbia, Vancouver, Canada

**Keywords:** Shared decision-making, Levonorgestrel intrauterine system, Tubal ligation, Salpingectomy, Tubal occlusion, Female, Permanent contraception, Sterilization

## Abstract

**Background:**

For women seeking permanent contraception, there are a variety of options available including surgical techniques such as tubal ligation or bilateral salpingectomy, in-clinic procedures such as hysteroscopic techniques using micro-inserts, or the levonorgestrel-releasing intrauterine contraceptive. Despite the various methods available for women who are seeking permanent contraception, there is not a review or decision-making tool that systematically brings together outcomes related to effectiveness, tolerability, adverse effects, non-contraceptive benefits, recovery, or accessibility: all of which are important for shared decision-making between patients and health care providers.

**Methods:**

We registered our protocol [on Prospero: CRD42016038254] following PRISMA guidelines. A search strategy was created in collaboration with a librarian, and three databases (EMBASE, PubMed, Web of Science) will be searched along with secondary screening of relevant articles. A third reviewer will adjudicate any discrepancies. Data will be extracted independently according to population, intervention, comparison, outcomes (PICOS); length of follow-up; and funding. Articles will be assessed for bias using the Newcastle-Ottawa Scale and the Cochrane Collaboration tool. If appropriate, a network meta-analysis will be conducted to rank and analyze each method according to each objective. If heterogeneity between studies is too high or it is not possible to conduct a network meta-analysis, a narrative analysis of the study results will be provided.

**Discussion:**

Clinicians and their patients seeking permanent contraception have several options, yet we were unable to find a systematic review or decision support tool helping to facilitate shared decision-making. This systematic review can inform patients, providers, and health policy decision-makers about which options of permanent contraception will meet different reproductive goals according to various outcomes, which can lead to better health, social, economic, and mental well-being for reproductive age women. This can also aid our understanding of resulting costs to the health care system.

**Systematic review registration:**

PROSPERO CRD42016038254

## Introduction

### Rationale

Continuing high rates of unintended pregnancy in Canada suggest that there is a discrepancy between available methods of contraception and contraception use and compliance. Literature suggests that when patients are involved in treatment plans through shared decision-making, there is better adherence and thus better outcomes [1]. Therefore, there is a significant need to involve women in choosing an appropriate method of contraception through shared decision-making, so that they are matched with a method that meets their and their families’ reproductive goals. For some women who are finished having children or who do not wish to have children, permanent contraception or long-term contraception methods may be an appropriate choice.

Trends worldwide and across North America illustrate growing numbers of women utilizing permanent contraceptive methods [2]. In the USA, approximately 290,000 cases of interval sterilization occur annually, and permanent contraception is now used by 25% of women of reproductive age (15–44 years) in the USA who are using contraception [3]. While permanent contraception continues to be a popular method of choice among women who do not wish to have children, recent trends have shown an increase in the use of the intrauterine device (IUD) across almost all populations of women using contraception in the USA [4]. Similarly in Canada, permanent contraception is the fourth most commonly used method among women who use contraception [5].

There are several options available for women seeking permanent contraception. Laparoscopic tubal ligation and hysteroscopic tubal occlusion are among the most common methods of surgical permanent contraceptive methods [6]. Laparoscopic tubal ligation is a surgical procedure in which fallopian tubes are cut, sealed, or burned with an electrical current through a small abdominal incision, preventing the movement of the egg from the ovary to the uterus for fertilization and implantation. The procedure is done under a general or local anesthetic and can be done immediately following a vaginal or cesarean delivery [7]. Approximately 5.5 pregnancies will occur out of every 1000 tubal ligations performed [8].

Hysteroscopic tubal occlusion (commonly known as Essure® or Adiana®) is a surgical procedure in which small metal clips or “microinserts” are inserted into the fallopian tubes with a catheter and held in place by stainless steel inner and nickel-titanium outer coils. These coils encourage tissue growth, which after approximately 3 months occludes the fallopian tubes, preventing sperm from reaching the egg and the egg from reaching the uterus. Placement of the clips is typically performed using a hysteroscopic approach, under local anesthetic [9]. It takes approximately 3 months for occlusion to occur. During this time, a woman is required to use alternate contraceptive methods. A post-procedure confirmation through a hysterosalpingogram (HSG) exam is required before a woman can discontinue alternative methods and the procedure is considered complete [10]. In an HSG exam, iodine-based dye is placed through the cervix, and X-rays are taken to determine full occlusion of the fallopian tubes, thus completion of the procedure. Failure rates are reported to be between 0.2 and 0.5% based on results after a successful HSG exam [11].

In British Columbia, Canada, recent trends regarding permanent contraception are shifting towards the use of the bilateral salpingectomy. The increased frequency of salpingectomy is largely due to emerging theoretical evidence suggesting that epithelial ovarian cancers may originate from the fallopian tube, and removing the fallopian tubes may play a role in preventing ovarian cancer [12]. A British Columbian regional cancer initiative in 2012 encouraged surgeons to consider performing an opportunistic salpingectomy in place of tubal ligation for women seeking permanent contraception. The regional ovarian cancer initiative proposing the increase in uptake of bilateral salpingectomy for prophylactic reasons has stemmed a debate on whether the procedure should be adopted based on the theoretical evidence of a reduction in ovarian cancer or whether physicians are obligated to wait until there is sufficient evidence. The debate centers around whether salpingectomies should be considered alongside other methods of permanent contraception such as tubal ligation or whether it should be performed within the context of a clinical trial as a means of gathering evidence first [13]. With a significant uptake of bilateral salpingectomies being performed for permanent contraception (from 0.5% in 2011 to 33% in 2015 in British Columbia alone), it is important to understand how the procedure compares to and fits alongside other available options [12, 14].

Additionally, long-acting reversible contraceptives may also be appropriate for women seeking permanent or long-term contraception. There are two types of intrauterine devices which act as long-acting, reversible methods of contraception available for women in North America: Copper T intrauterine device (IUD) and the levonorgestrel-releasing intrauterine contraceptive (LNG-IUC). The Copper T-IUD is a copper-wrapped, polyethylene T-shaped device. Release of copper into the uterine cavity creates a foreign-body reaction resulting in an inhabitable environment for sperm transport or fertilization [15]. The LNG-IUC is also a polyethylene T-shaped device, infused with 52 mg of levonorgestrel instead of copper. As levonorgestrel is administered into the uterine cavity, uterine lining is thinned and at the same time cervical mucous thickens to block sperm entry. While both intrauterine devices are effective, the LNG-IUC has a higher effectiveness rate at 0.1 compared to 0.8 pregnancies per 100 women [15]. With a higher rate of pregnancy prevention than tubal occlusion, the LNG-IUC is underutilized in conversations about permanent contraception and arguably should be included in the decision-making process when considering methods of permanent and/or long-acting contraception [16].

With new and innovative methods of permanent contraception being introduced, it is important that health policy and guidelines for contraceptive techniques and methods reflect current evidence. Women should be counseled on all available options, including the associated potential risks and benefits, so as to make the best decision to meet their specific reproductive goals. While there is a range of options available to women seeking permanent and/or long-term contraception, there yet remains to be a decision support tool or a systematic review comparing available evidence for existing options. We aim to perform a systematic review and network meta-analysis comparing hysteroscopic tubal occlusion (HTO), laparoscopic tubal ligation (LTL), bilateral salpingectomy (BS), and the levonorgestrel-releasing intrauterine contraceptive (LNG-IUC) on effectiveness, adverse effects, tolerability, and non-contraceptive benefits for women, and health system cost-effectiveness in high-resource countries.

### Objectives

This research aims to fill a gap in the literature comparing the different methods of reversible long-acting and/or permanent contraception available and, in doing so, inform current policy guidelines regarding female permanent contraception alternatives. This can have further implications for areas that do not have the capacity to perform invasive or surgical techniques, providing non-surgical alternatives for women seeking permanent contraception. Understanding the associated relative risks and benefits has the ability to facilitate shared decision-making between patients and physicians, as a way to best meet their patients’ needs and values.

Specific aims include:

1. Assessing the clinical risks and benefits of four contraceptive methods for women seeking permanent contraception: tubal occlusion, tubal ligation, LNG-IUC, and opportunistic salpingectomy;
2. Use this information and existing literature to compare these four methods according to effectiveness, adverse effects, tolerability, non-contraceptive benefits, patient recovery, and accessibility;
3. Create tools that will be useful to inform health policy and clinical guidelines, and promote shared decision-making among patients and physicians

### Population

This systematic review is focused on reproductive age women (ages 15–49 years) living in high-resource countries as defined by the World Bank Country and Lending Groups [17] who are seeking permanent contraception.

Intervention/comparison:

- Hysteroscopic tubal occlusion with microinserts
- Laparoscopic tubal ligation
- Levonorgestrel-releasing intrauterine contraceptive
- Bilateral salpingectomy

Primary outcomes:

- Effectiveness is defined as the typical use rate at which the method prevents pregnancy: the inherent efficacy of the contraceptive method, and the correct application [1]. It may be reported as a percentage, or number of pregnancies reported per sample size.
- Adverse effects are defined as an undesired harmful effect resulting from the method of contraception being employed—hysteroscopic tubal occlusion, laparoscopic tubal ligation, LNG-IUC, and opportunistic salpingectomy. Adverse effects include but are not limited to prolonged pain in lower abdomen; minor complications such as infection and wound separation; and major complications including heavy blood loss, anesthesia problems, injury during surgery/insertion, need for larger incision.
- Tolerability is defined as the ability of a patient to withstand any adverse effects and will be measured as the number of women who successfully used the procedure/treatment and did not require removal of the LNG-IUC/microinsert clips or additional procedures to attend to any adverse effects that may have resulted from the respective procedure.
- Non-contraceptive benefits are defined as effects that are positive for a woman apart from effectiveness at preventing pregnancy including, but not limited to, decreased risk of ovarian cancer, alleviation of acne, relief of dysmenorrhea and/or menorrhagia, or decreased associated infections or diseases such as pubic inflammatory disease (PID).
- Patient recovery is defined as the time reported for a woman to recover, or to return to her typical state of well-being, after the procedure. Patient recovery includes, but is not limited to, post-procedure pain reported, reported satisfaction, and recovery time, including length of hospital stay.
- Accessibility of each treatment is measured in terms of the out of pocket costs for the procedure, eligibility or ineligibility for each procedure, wait times, and the locations where the procedure can be performed (i.e., hospitals, clinics).

Secondary outcomes include:

- Length of the procedure
- Number of follow-ups needed to ensure that the method is complete or is required for safety monitoring
- Who is eligible or not eligible for each respective method
- Cost to health care system for each respective method

## Methods

Preferred Reporting Items for Systematic Reviews and Meta-Analyses (PRISMA) guidelines will be followed [18].

### Eligibility criteria

Inclusion criteria included:

- Studies involving samples of women of reproductive age (15–49 years)
- Study designs include case-control, comparative observational studies (retrospective and prospective), and single or multi-centered randomized controlled trials evaluating pairwise comparisons related to any of the four methods involved in the review (hysteroscopic tubal occlusion, laparoscopic tubal ligation, levonorgestrel-releasing intrauterine contraceptive, bilateral salpingectomy, and/or a control)
- Studies evaluating outcomes related to effectiveness, adverse effects, tolerability, non-contraceptive benefits, patient recovery, and financial accessibility as defined above
- Studies performed in high-income countries as defined by the World Bank Country and Lending Groups [17]
- All articles must be peer reviewed and published in English.
- No date restrictions.
- Electrocoagulation, tubal rings, Filshie clips, and the Pomeroy technique are all considered as “tubal ligation” as several included studies did not differentiate between which laparoscopic technique was used. Essure, Adiana, and surgical contraception techniques using microinserts were combined into one treatment under ‘tubal occlusion.’ Bilateral salpingectomy, opportunistic salpingectomy, prophylactic salpingectomy, and tubectomy were combined under “salpingectomy.”

### Information sources and search

Relevant articles were identified through a search of EMBASE, Web of Science, and PubMed (Medline) using a combination of MeSH terms and key words related to hysteroscopic tubal occlusion, laparoscopic tubal ligation, levonorgestrel-releasing intrauterine contraceptive (LNG-IUC), and opportunistic salpingectomy after consultation with a reference librarian familiar with systematic reviews. Copies of the search strategies can be found at http://med-fom-cart-grac.sites.olt.ubc.ca/files/2016/05/Search-Strategies-Librarian-edit.docx.

### Study records

#### Screening for eligible studies

Throughout the review process, articles will be stored in the Mendeley Desktop Manager (Version 1.17.17, Mendeley Ltd., 2017) [19]. Duplicates will be removed using the duplication tool. Reviewers RG and BV will review titles and abstracts, and obtain full text for any articles deemed to be relevant. Reviewers will then apply the inclusion criteria to come up with a list of eligible articles. All studies, which upon inspection of the full text do not merit inclusion, will be detailed in an “Excluded Studies” table with reasons for exclusion. Any disagreements will be adjudicated by author WVN.

#### Data extraction and management

RG created the initial data extraction form. The data extraction form will then be pilot-tested on a randomly selected subset of studies and will be modified according to feedback and outcomes. This will ensure that the data extraction form is providing a comprehensive method of collecting key findings for this review. Data will be extracted from each study to meet the inclusion criteria including population, intervention, comparisons, outcomes, and study design (PICOS); follow-up period; biases; and funding source. Reviewers RG and BV will perform data extraction independently, and discrepancies will be discussed and consulted with third reviewer WVN.

### Risk of bias in individual studies

Each individual study will be evaluated for risk of bias according to the Newcastle-Ottawa Scale (NOS) [20] for assessing the quality of non-randomized studies in meta-analyses, and the Cochrane risk of bias tool [21] for randomized controlled trials with a focus on study design, sampling strategy, appropriateness of analytical methods used, and other biases that may be present. The NOS tool is a widely used and well-established tool for assessing the risk of bias in observational studies and has been previously validated for case-control and longitudinal studies [20]. The Cochrane tools are domain-based evaluations rather than a scaled or checklist evaluation. Assigning numbers and weighting different items on a scale can be difficult to justify: domain-based adjudication allows for critical assessment and a further interrogation into internal validity, and the extent to which inclusion and exclusion criteria were clearly described and implemented [21]. The risk of bias for each included article will be presented in a “Risk of Bias” table according to Ottawa-Newcastle and Cochrane standards.

### Data synthesis

If appropriate, a network meta-analysis will be conducted using statistical software following the Cochrane Methods network meta-analysis toolkit. The study data will be quantitatively synthesized by computing direct estimates between comparisons, indirect estimates for comparisons, pooling the indirect and direct comparisons to create the mixed estimate for comparisons, and checking the consistency between the direct and indirect estimates following the Bucher method [22]. Baseline characteristics, weighted means, successful placement or successful outcome, and odds ratio (OR) or risk ratio (RR) according to individual sample size will be used. *I*^2^ (95% confidence interval) statistic for each meta-analysis will be estimated to assess heterogeneity across studies. As per the Cochrane Handbook, we will consider heterogeneity to be low if *I*^2^ = 0–25%, moderate if *I*^2^ = 25–50%, and substantial if *I*^2^ > 50% [21].

Data will be summarized by abstracting relevant data, developing data tables, and synthesizing the literature. The feasibility of completing a quantitative analysis (network meta-analysis) will depend on the available literature, conceptual homogeneity, and completeness of reporting results that are necessary for completing a quantitative analysis. In the case that heterogeneity between studies is too high or is not possible to conduct a network meta-analysis, we will provide a narrative analysis of the study results. Patterns of effects, similarities, and differences between studies will be presented according to the Cochrane Consumers and Communication Review Group guidelines [23]. To maintain transparency about selective reporting, any deviations from our protocol will be reported in the final review under a “differences between protocol and review section” with explanations for any deviation.

#### Presenting and reporting results

Preferred Reporting Items for Systematic Reviews and Network Meta-Analyses (PRISMA) statement will be followed [24] including PRISMA “diagram to illustrate the process of selecting eligible studies.”

## Discussion

Previous research has provided updates on different options of methods of permanent contraception; however, to date, there is not a study that systematically brings together the major methods of permanent contraception in a comparison format with other methods of permanent and/or long-acting forms of contraception. A systematic review analysis comparing the benefits and risks of hysteroscopic tubal occlusion using microinserts, laparoscopic tubal ligation, the LNG-IUC, and opportunistic salpingectomy for reproductive-aged women seeking permanent and/or long-acting contraception will provide an evidence-based tool to inform regional health guidelines in contraceptive counseling and contraceptive methods. The document resulting from this research will inform patients, providers, and health policy decision-makers about which methods of permanent contraception will meet different reproductive goals, according to various outcomes.

## Abbreviations

BS: Bilateral salpingectomy
EMBASE: Excerpta Medica database
HSG: Hysterosalpingogram
HTO: Hysteroscopic tubal occlusion
LNG-IUC: Levonorgestrel-releasing intrauterine contraceptive
LTL: Laparoscopic tubal ligation
MeSH: Medical Subject Headings
